# A biomedical educational intervention to change explanatory models of psychosis among community health workers in South India

**DOI:** 10.4103/0019-5545.31575

**Published:** 2006

**Authors:** D. Joel, M. Sathyaseelan, R. Jayakaran, C. Vijayakumar, S. Muthurathnam, K.S. Jacob

**Affiliations:** * Lecturer in Psychiatric Nursing, College of Nursing, Christian Medical College, Vellore 632004; ** Reader in Psychiatric Nursing, College of Nursing, Christian Medical College, Vellore 632004; *** Professor of Community Nursing, College of Nursing, Christian Medical College, Vellore 632004; **** Professor of Community Nursing, College of Nursing, Christian Medical College, Vellore 632004; ***** Senior Statistician, Department of Biostatistics Christian Medical College, Vellore 632002, Tamil Nadu; ****** Professor of Psychiatry, Christian Medical College, Vellore 632002, Tamil Nadu

**Keywords:** Community health aides, mental disorders, education

## Abstract

**Background:**

Community health workers in developing countries commonly hold indigenous beliefs about mental illness which differ markedly from biomedical models.

**Aim:**

To test the effect of a biomedical intervention on explanatory models (EMs) of community health workers.

**Methods:**

Indigenous beliefs about chronic psychosis were elicited from community health workers. The Short Explanatory Model Interview formed the basis of the interview. Half the workers were taught about the biomedical model after discussing their EMs of chronic psychosis. The others did not receive education. The beliefs of all community health workers were reassessed 2 weeks after the initial assessment.

**Results:**

A variety of indigenous beliefs, which contradicted the biomedical model, were elicited at the baseline evaluation. Seeking biomedical help at follow up was significantly related to receiving education about the biomedical aspects of chronic psychosis (OR 17.2; 95% CI: 18.75, 15.65; p<0.001). This remained statistically significant (OR 9.7; 95% CI: 82.28, 1.14; p<0.04) after using logistic regression to adjust for baseline variables.

**Conclusion:**

The high prevalence of non-medical beliefs among community health workers suggests the need to elicit and discuss beliefs before imparting knowledge about biomedical models of mental disorders. Biomedical educational intervention can change EMs of mental illness among health workers.

## INTRODUCTION

Local culture and beliefs influence many aspects of human behaviour such as idioms of distress, help-seeking, treatment compliance, patient satisfaction and coping. Perspectives of mental illness, also called explanatory models (EMs), play an important role in health-related behaviours and in patient–health worker interaction.^1^–^5^ Diverse explanations are offered to explain mental illness including social circumstances, relationship difficulties, witchcraft or sorcery, or a broken taboo.^5^ Eliciting local EMs in routine clinical psychiatric practice gives a better understanding of the subjective experience of illness,^1^–^5^ attitude towards and compliance with treatment, and thus promote therapeutic adherence and improve clinical outcomes.

Despite the past few years having witnessed an increase in literature regarding local beliefs about causes of schizophrenia,^6^–^10^ there is paucity of studies evaluating local EMs and the efficacy of intervention to change EMs.^11^ Consequently, there is a need to understand not only the local perspectives but also to evaluate the efficacy of inter-ventions based on biomedical models of schizophrenia.

Understanding local perspectives is crucial for establishing culturally sensitive healthcare for a community. Community health workers are part of the World Health Organization's plan to integrate mental health component into primary healthcare in developing countries.^12^–^15^ However, many community health workers hold indigenous beliefs about mental illness which differ markedly from the biomedical models emphasized by mental health professionals.^16^ Most education programmes have not evaluated local perspectives and the role of training in changing indigenous beliefs held by health workers. This study attempted to evaluate a structured intervention programme used to educate health workers about the biomedical aspects of psychosis in changing indigenous beliefs.

## METHODS

### The setting

The Rural Unit for Health and Social Affairs (RUHSA), a community health programme of the Christian Medical College Hospital, Vellore, India, serves the K.V. Kuppam Block in the Vellore district in the state of Tamil Nadu. The block has an area of 180 sq. km. The programme operates in all 84 villages in the area. The total population served is about 120,000. The majority of the population speaks Tamil, is involved in agriculture and animal husbandry and belongs to the lower socioeconomic strata.

The frontline of RUHSA's healthcare structure is the Community Health Worker. The health worker is supported by the community health team (consisting of a doctor, nurse, community organizer and a health aide), which visits every village every week. Cases requiring greater medical attention are referred to the base hospital. Morbidity and mortality data, birth and death statistics are also reported.

### The sample

The details of the study were explained to the community health workers working in the programme and informed consent was obtained. Eighty (88.9%) workers, from a total of 90, volunteered for the study.

### Baseline evaluation

The Short Explanatory Model Interview^17^ formed the basis of the evaluation. Kleinman's original concepts,^1^ which examined health and sickness from an anthropological perspective, form the basis of the interview. The interview explores *emic* perspectives of illness.^17^ It employs open-ended questions and is semi-structured. The language is simple and does not include any medical or technical words or phrases. The subjects are encouraged to talk openly about their attitudes and experience with the aim of eliciting concepts held, and relationship to current situation and culture. Probes are also employed to confirm the concepts mentioned and to explore areas which the patients did not volunteer.

The interview is divided into five sections to cover the subject's background, nature of presenting problem, help-seeking behaviour, interaction with physician/healer, and beliefs related to mental illness. The section on the subject's background emphasizes individual and cultural aspects. Basic demographic data, interpersonal relationships, housing, work, social life, religion, life history and specific cultural beliefs are assessed. An individual's beliefs related to the nature of the presenting problem are examined in detail and include the reason for consulting, name of the problem, perceived causes, consequences, severity and its effects on the body, emotion, social network, home life and on work. *Emic* symptoms are elicited by open-ended probing. Help-seeking behaviour, especially contact with alternative non-medical sources (e.g. traditional healers) are also examined. The details of the interaction with the physician/healer are also evaluated in terms of expectation and satisfaction.

The section on illness beliefs consists of three vignettes of common mental disorders. The vignette employed for the study describes the problems faced by individuals with schizophrenia.^16^ It was followed by open-ended questions to elicit the individual's attitudes concerning the clinical problem and attempt to enquire as to whether the subject considers the presentation as a problem and also whether there is an illness. This was followed by open-ended questions to elicit the health worker's beliefs concerning the clinical problem, perceived causes, consequences, severity and its effects on body, emotion, social network, home-life and on work, severity, possible course of action, help-seeking behaviour and the role of the physician/healer.

Each section is designed to stand-alone and this allows the interviewer flexibility in the order of questioning. It also allows for focus on certain aspects of the interview and the omission of others aspects depending on the overall objectives of the study. The SEMI takes about 20–30 minutes to complete.

This interview explored *emic* perspectives of illness. The language was simple and did not include any medical or technical words or phrases. Probes were also employed to confirm the concepts mentioned and to explore areas, which the health workers did not volunteer. A verbatim record of the responses was made. The responses were later grouped into categories using the recommended procedure. The Tamil version of the instrument^16^,^18^ was employed. The instrument was administered to each health worker individually at the health centre.

### The intervention

The community health workers were divided into two groups depending on the geographical location of their villages and peripheral health centres. The geographical distance between the two groups was to prevent workers who were educated from discussing the educational material with subjects who were in the control group. Forty workers were taught about the biomedical aspects of schizophrenia. The training programme discussed local beliefs about mental illness, elicited by the SEMI interview. The health workers were encouraged to discuss their beliefs and their implications. The structured teaching programme, of 2-hour duration, then described the symptoms, causes, treatment and referral.^19^ The training was given in batches of 6–7 workers. The 40 workers of the control group were not educated during the study period.

### The follow up

All the subjects were re-interviewed using the same instrument after a period of 2 weeks. The instruments were administered to each worker individually.

### Analysis

The qualitative data generated by the Tamil version of the SEMI were examined. Items were enumerated and the broad facets identified. Items, which occurred frequently, were allocated independent numerical codes. The procedure employed for this was similar to that used for the original SEMI version.^17^ Subsequently, SEMI data for all the subjects were analysed and *emic* items coded dichotomously (not reported/reported). This allowed for quantitative analysis. The following analysis was done: (i) comparison of baseline socio-demographic and EMs between groups, (ii) change in EMs after 2 weeks. Mean and standard deviation were employed to describe continuous variables, while frequency distributions were obtained for categorical data. The chi square test was used to assess the significance of associations for categorical variables. Student *t*-test was used to test the associations for continuous data. Logistic regression was done to exclude confounding. Odds ratios and confidence intervals were calculated. The statistical software SPSS for Windows Release 6.1.3 was employed for the analysis.

## RESULTS

### Sociodemographic characteristics of the sample

Eighty community health workers (88.9%) volunteered to take part in the study. Their mean age was 48.15 (SD 7.64) years. All were women. The majority were married (74; 92.5%) and had received at least primary school education (43; 53.8%). The average number of years of experience as a community health worker was 14.87 (SD 4.87) years. Ten health workers did not take part in the study as they were on leave during this training programme. Those who did not take part in the training did not differ from those who participated in the study on the following variables: age, gender, marital status, years of schooling and work experience.

### Baseline EMs and comparison between groups

Table 1 documents the sociodemographic characteristics and EMs at baseline for the two groups. The difference between the two groups in terms of gender, age, marital status, education and number of years of experience as a health worker was not statistically significant. A minority of the subjects (in the whole sample) identified the problems described in the vignette as psychological (37; 46.3%), considered it a disease (28; 35%), attributed it to black magic (32; 40%) or evil spirits (23; 28.8%), felt that help should be sought from a nurse/doctor/hospital (19; 23.8%) or traditional healers (9; 11.3%) and the temple (14; 17.5%). The majority also attributed the problems to economic difficulties (42; 52.5%). Seventy subjects (87.5%) in the whole sample held at least one non-biomedical explanation for psychosis (e.g. black magic, evil spirits as cause, non-disease concept, seeking treatment from traditional healers or temples and not seeking medical help). However, the differences between the baseline sociodemographic variables and EMs of the experimental and control groups were not statistically significant (Table 1).

**Table 1:** Comparison of sociodemographic variables and explanatory models of psychosis at baseline

Characteristic	Experimental group (*n*=40) *n* (%)	Control group (*n*=40) *n* (%)	Statistical significance
Female gender	40 (100)	40 (100)	NS*
Age (in years)	Mean 49.57 (SD 6.6)	Mean 46.73 (SD 8.4)	NS**
Marital status: Married	37	37	NS*
Received formal education	19	24	NS*
No. of years of experience as health worker	Mean 14.80 (SD 5.0)	Mean 14.95 (SD 4.8)	NS**
**Details of explanatory models**			
Vignette suggests psychological problems	22 (55)	15 (37.5)	NS*
Vignette suggests disease	15 (37.5)	13 (32.5)	NS*
Problems caused by black magic	16 (40)	16 (40)	NS*
Problems caused by evil spirits	15 (37.5)	8 (20)	NS*
Problems due to poverty	18 (45)	24 (60)	NS*
Visit a nurse/doctor/hospital for treatment	10 (25)	9 (22.5)	NS*
Doctor cannot help subject	10 (25)	9 (22.5)	NS*
Nurse/doctor should prescribe medication	5 (12.5)	6 (15)	NS*
Subject should take medication	14 (35)	8 (20)	NS*
Visit traditional healers for treatment	7 (17.5)	2 (5)	NS*
Pray and visit the temple for cure	4 (10)	10 (25)	NS*
Any non-biomedical belief	33 (82.5)	37 (92.5)	NS*

* ÷^2^ test for significance of association;

** Student *t*-test; NS Not statistically significant

### Comparison of EMs at follow up

Table 2 shows the EMs of the health workers at 2 weeks after education/baseline assessment. The experimental group, which had received education, showed a statistically significant difference in EMs of psychosis in comparison with the group of health workers who did not receive education. A significant proportion of the EMs of health workers who were taught about schizophrenia had changed in favour of biomedical explanations. The only aspect that did not change significantly was the belief that psychosis was due to poverty.

**Table 2:** Comparison of explanatory models of psychosis after 2 weeks

Details of explanatory models	Experimental group (*n*=40) *n* (%)	Control group (*n*=40) *n* (%)	Statistical significance* (p value)
Vignette suggests psychological problems	38 (95)	22 (55)	<0.001
Vignette suggests disease	40 (100)	11 (27.5)	<0.001
Problems caused by black magic	2 (5)	21 (52.5)	<0.001
Problems caused by evil spirits	0 (0)	6 (15)	<0.02
Problems due to poverty	16 (40)	20 (50)	NS
Visit a nurse/doctor/hospital for treatment	38 (95)	21 (52.5)	<0.01
Doctor cannot help subject	0 (0)	6 (15)	<0.03
Nurse/doctor should prescribe medication	24 (60)	10 (25)	<0.002
Subject should take medication	36 (90)	11 (27.5)	<0.001
Visit traditional healers for treatment	0 (0)	5 (12.5)	<0.03
Pray and visit the temple for cure	0 (0)	4 (10)	<0.03
Any non-biomedical belief	6 (15)	36 (90)	<0.0001

* ÷^2^ test for significance of association; NS Not statistically significant

Seeking biomedical help at follow up was significantly related to receiving education about the biomedical aspects of chronic psychosis (OR 17.2; 95% CI: 18.75, 15.65; p<0.001). This remained statistically significant (OR 9.7; 95% CI: 82.28, 1.14; p<0.04) after using logistic regression to adjust for the following baseline variables: age, education, years of experience as a health worker, disease concept, belief in black magic and evil spirits, seeking help from temples and traditional healers.

## DISCUSSION

This study attempted to evaluate the role of training in changing EMs of chronic psychosis among community health workers employed in a comprehensive community health programme in rural South India. It discussed local beliefs and presented alternative biomedical explanations for schizo-phrenia. The study attempts to combine qualitative and quantitative methodologies. It uses the SEMI which is designed to combine such methodology for use in field work. While it may not meet rigorous ethnographic standards, it is an attempt to look at qualitative data from an epidemiological point of view. The specific limitations of the study were that the intervention was not randomized and the assessment of outcome was not done blind to the intervention status. In addition, long-term retention of learnt information and practice were not evaluated. However, the two groups were comparable on baseline evaluation and these factors were adjusted for in the multivariate analysis. Moreover, the use of the SEMI and the verbatim recording of EMs would have helped in eliciting unbiased information.

The baseline assessment done for this study revealed a wide variety of local beliefs regarding mental illness.^16^ Many of these beliefs contradicted the biomedical model of psychosis. These beliefs were not directly challenged during the training but the subjects were encouraged to consider biomedical alternatives. The nature and extent of indigenous beliefs suggest the need to explore and discuss them prior to training. The results of the study showed that change in EMs was possible with training. However, the belief that poverty is a cause of psychosis was difficult to change and may require greater input to alter. Some of the changes in traditional beliefs in the experimental group were marked with all subjects claiming to hold the biomedical model. The non-blind assessment of beliefs having contributed to ‘socially desirable responses' among some of the participants cannot be ruled out.

Non-biomedical beliefs about schizophrenia can delay early recognition of disease, prevent early institution of medication, interfere with medication compliance and follow up, resulting in poor outcome. Local beliefs of community health workers and other health professionals need to be elicited and discussed before presenting alternative biomedical explanations about illness. However, most training programmes for community health workers^12^–^14^ have not examined local beliefs and their change with education. The wide variety of non-medical beliefs related to mental disorders in developing countries mandates the need to examine locally held perspectives about illness during training for health workers.

## CONCLUSION

Beliefs, which directly contradict the biomedical model, are commonly found among the general population and among community health workers, especially in non-western cultures. Such beliefs have to be elicited and discussed before the teaching of biomedical models. The results of the study suggest that teaching programmes can alter EMs of community health workers. However, the persistence of changes in beliefs and its effect on practice needs to be confirmed.
